# The True Price of External Health Effects from Food Consumption

**DOI:** 10.3390/nu15153386

**Published:** 2023-07-30

**Authors:** Felix Seidel, Benjamin Oebel, Lennart Stein, Amelie Michalke, Tobias Gaugler

**Affiliations:** 1 Faculty of Mathematics, Natural Sciences, and Materials Engineering, University of Augsburg, 86159 Augsburg, Germany; felix.seidel@uni-a.de; 2 Faculty of Business Administration, Nuremberg Institute of Technology, 90489 Nuremberg, Germany; tobias.gaugler@th-nuernberg.de; 3 Faculty of Mathematics and Natural Sciences, University of Greifswald, 17489 Greifswald, Germany; lennart.stein@uni-greifswald.de (L.S.); amelie.michalke@uni-greifswald.de (A.M.)

**Keywords:** health costs, food consumption, nutrition and dietetics, public health, family health, sustainable development, true prices, externalities

## Abstract

Although global food consumption costs more in terms of impact on human life than money is spent on it, health costs have not been consistently quantified or included in food prices to date. In this paper, a method to determine the external health costs of nutrition and dietetics is developed by employing the cost-of-illness (COI) and true cost accounting (TCA) approaches. This is done exemplarily for the reference country Germany. The results show that 601.50 € per capita and 50.38 billion € in total external health costs are incurred annually due to nutrition. Overall, most costs are accrued through excessive meat consumption (32.56% of costs), deficient whole grain intake (15.42% of costs), and insufficient uptake of legumes (10.19% of costs). Comparing the external health costs with the external environmental costs in Germany, it can be seen that of the total annual costs of around 153.86 billion €, 67.26% originate from environmental impacts and 32.74% from impacts on human life. In order to achieve the 17 Sustainable Development Goals and to increase family as well as public health, there is a need to internalise these external costs into actual food prices.

## 1. Introduction

### 1.1. The Potential Impact of Externalities on Food Prices

Intense droughts, water scarcity, severe fires, rising sea levels, melting polar ice, and declining biodiversity are just a few examples of the consequences of global climate change. Since the 1800s, humans have been the main reason for these extreme events [1]. The 2030 Agenda for Sustainable Development has provided countermeasures to that by defining 17 Sustainable Development Goals (SDGs) for the global community to advance socially, economically, and environmentally sustainable development [2]. The SDGs aim to improve health and education, reduce inequalities, and boost economic growth [3,4]. As one of the main emitters of especially greenhouse gases (GHG emissions), the agricultural sector globally represents one of the main drivers of climate change [5]. Thus, a distinction between farming systems and food categories must be undertaken. Occurring air pollutants also contribute to respiratory disease from smog [6]. Emissions, therefore, not only have an impact on the environment, but also on human life. The resulting costs are not included in the market prices to date and are, therefore, not compensated for [7].

GHG emissions represent an example of an externality, which is defined as any action that affects the welfare or available opportunities of an individual or group and is neither directly paid for nor compensated [8] (pp. 263–283). Thus, actors who are not directly involved in their creation ultimately bear the burden of externalities [9]. Externalities represent a side effect of an economic activity, the costs of which are not part of the prices paid by consumers or producers [7]. Consequently, externalities have no economic currency and lead to a distortion of the market [8] (pp. 263–283). As a result, no incentives for healthier eating are set, for example [10]. In relation to market failures, one speaks of technological or physical externalities. Another form is pecuniary or price-effect externalities, which help some groups and harm others, but do not necessarily represent market failure. A further subdivision is made by distinguishing between positive or beneficial and negative or harmful externalities [8] (pp. 263–283).

In this paper, only technological externalities are considered, which are documented in statistics on mortality and diseases.

Various types of externalities exist. Regarding food, environmental, social, health, and economic externalities are distinguished [10]. It is, however, difficult to look at the individual species in complete isolation.

A study by the Scientific Group of the United Nations (U.N.) Food Systems Summit shows that food externalities amount to USD 20 trillion per year, which corresponds to more than twice the global food consumption. While USD seven trillion is the result of environmental costs, the bulk of the costs of USD eleven trillion is attributable to impacts on human life (human capital). Of this, an average of 57% is due to direct healthcare costs and 43% to indirect costs such as informal care and losses in labour productivity. The high proportion of unpriced health externalities makes sustainable and healthy food less affordable for consumers and less profitable for producers than comparatively unhealthier alternatives; hence, representing one main reason for global malnutrition. For example, 690 million people were undernourished in 2019, and ten million die every year due to unhealthy eating habits. Global nutrition guidelines could save five million lives and 79 million life years per year. Moreover, according to this study, up to USD 735 billion in health costs could be saved if such guidelines were introduced [10].

The COI approach was used to calculate the potential cost savings due to healthier diets. COI studies aim to identify and measure all the costs of a disease [11] by considering the direct as well as the indirect and intangible components [12]. They estimate the burden of a specific disease to society and, therefore, the savings that would be possible if the disease were to be eradicated [13] (pp. 143–148). COI analyses thus provide a measure of the size of a problem [14]. This approach is used in this paper to develop a methodology for calculating the health costs of specific diseases.

### 1.2. Calculating Health Costs

#### 1.2.1. Challenges in Determining Health Costs

In the scientific literature, no concrete, calculated health premiums or discounts for certain foods exist. The reason for this absence is both the difficulty to measure food products externalities in general as well as large uncertainties. The latter is due to incompletely captured fields of influence as well as fundamental uncertainties in the primary literature, in trade datasets, in modelling the strands of influence, and in monetising external costs. Specifically, estimates of health costs from food vary due to differing approaches and assessment methods, as well as inconsistent standards and guidelines [15], as this is an emerging field of research [10].

Furthermore, data sources and data collection in combination with the high complexity of the underlying value chains represent a key challenge. One of the main reasons why health and social components have not yet been sufficiently taken into account in corresponding approaches such as TCA to date is the main focus of companies on improving the natural capital base in impact assessment to improve shareholder value creation. The center of attention lies in reducing carbon emissions, as this is also a global focus, above all visible in the example of the 2030 Agenda. However, impacts on the oceans or workers’ health, for example, are hardly addressed. Therefore, a transdisciplinary approach is necessary to consider health and social consequences along the entire value chain [15].

The mentioned problem areas have the consequence that currently no uniformly quantified nutrition manual that can be utilised to determine concrete price premiums for the health effects of food has been published [10].

#### 1.2.2. Calculating Health Impacts

One solution to this problem is the Planetary Health Diet (PHD), which was designed for a global population of about 10 billion people in 2050. The basic ideas are a shift to healthy diets and sustainable food production [16] (pp. 447–492). Therefore, both human health requirements and planetary boundaries are considered [17] (pp. 14–21).

Furthermore, methods for internalising external costs already exist. One approach is TCA. Its first step is to define the basic framework. Then, one collects and prioritises externalities and, in the last step, identifies methods for quantification and monetisation [10,15,18,19]. TCA serves the purpose of making dependencies, flows, and negative as well as positive external effects visible. Although representing a new research method, TCA is already applied in advisory organisations to educate about environmental and social externalities, or by food companies to identify the need for action and, thus, optimise the resulting impacts in a targeted manner, for instance [10,15,19].

The TCA approach is in accordance with the U.N. SDGs as it represents a basis for the Polluter Pays Principle [19]. It says that the polluter must bear the costs of pollution prevention and control measures to make sure that the environment is in an acceptable state [20] (pp. 31–84). The approach has already made it possible to calculate the external climate costs of nutrition and thus draw attention to the inadequate pricing of animal products [7]. In addition, true food costs based on full life cycle analyses have already been determined [9], and specific climate and biodiversity costs from land-use change caused by animal-based products have been calculated [21].

Furthermore, statistics already exist on the direct and indirect health costs of common diseases such as CVD [22], T2DM [23,24,25,26,27], and cancer [28] (pp. 41–49). Data is also available for mortality and years of life lost due to disease or dietary risk factors [29,30].

Moreover, studies on aggregate global health costs [10] or on the health costs of individual countries such as the USA [15] or Switzerland [18] have been conducted. Besides, analyses often show how many external costs could be saved by following appropriate dietary guidelines or adopting an alternative dietary style. The latter is discussed in a paper by the Food and Agriculture Organization of the United Nations (FAO) in which the total expenses of diet are calculated. Thereby, wholesale, climate-change, and health costs are listed globally and by country, grouped by income. The health component is determined by indirect and direct costs for individual diseases that occur due to nutrition [31].

In another study by Springmann et al. (2018) [32], a tax on red and processed meat is calculated using the COI approach. In the first step, a reference consumption is established and an additional portion of red or processed meat is added to obtain disease associations. In the next step, the health costs per disease are determined via attributable deaths and the corresponding total health costs are calculated. Finally, the tax levels are ascertained, and the result of the methodology is evaluated. Thus, Springmann et al. (2018) [32] calculate a global average optimal tax of 0.28 USD/kg for red meat and 1.45 USD/kg for processed meat. For high-income countries, this is 0.94 USD/kg for red meat and 4.17 USD/kg for processed meat.

In summary, the vast majority of studies deal with the environmental costs of food and only a few focus on calculating the health costs of food. The COI approach is predominantly used for internalising health costs, in which direct and indirect costs of diseases are taken into account. Up to now, however, either the total global health costs have been calculated or only those for a group of countries, subdivided according to income. A specific health premium per kilogram was calculated for red and processed meat only, whereby here again a high level of aggregation was applied at the country level. To date, no methodology has been elaborated that determines the country-specific health costs of individual foods or their ingredients and, thus, creates reference values for potential price increases. Therefore, in this paper, we aim to develop a true pricing approach using the COI and TCA approaches to calculate the specific external health cost premiums of selected dietary risk factors for a reference country. Finally, different implementation options and their advantages and disadvantages will be discussed. Thus, this study answers the following research question: how high are the external health costs of food consumption?

## 2. Materials and Methods

### 2.1. Method Outline

In this section, we first describe the methodology to calculate health premiums, which represent external costs to date, for foods by dietary risk factors. In the first step, an approach is developed to quantify and monetise the quantity-standardised health costs of the harmful quantity for individual dietary risk factors. In the second step, the methodology is extended to include an approach for determining the concrete health premiums of individual products. Finally, we explain the calculation and sourcing of the input data.

### 2.2. Method in Short

**Figure 1:**
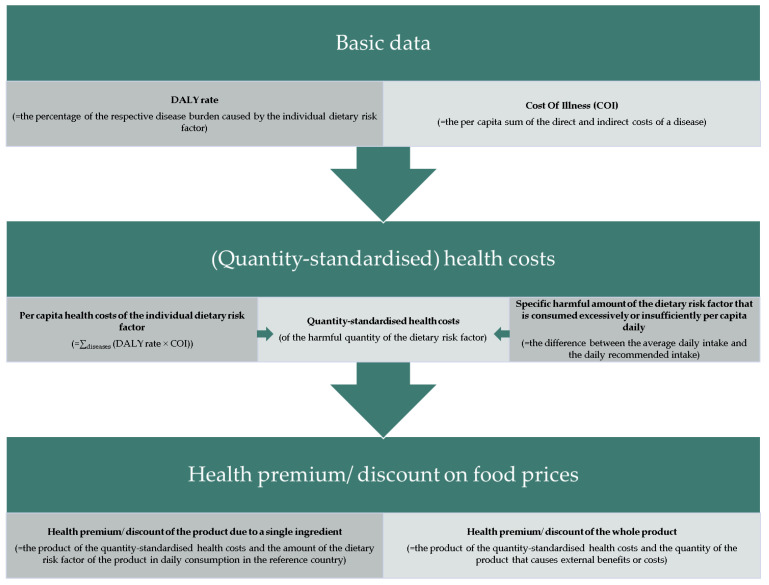
Abridged visualization of the process of the method.

**Figure 2:**
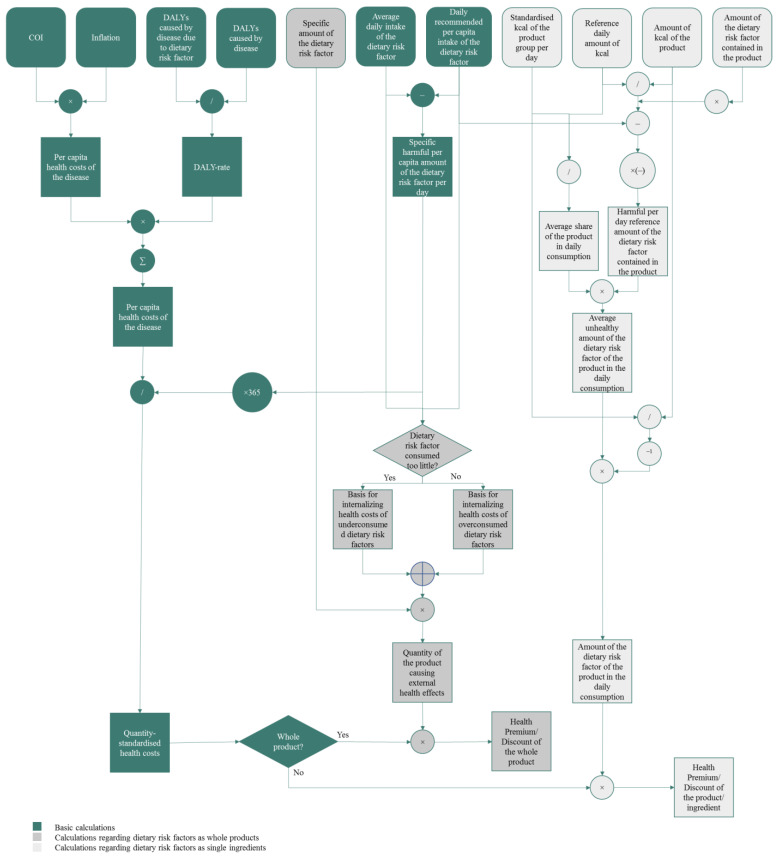
Detailed visualization of the process of the method.

Before starting the method, a reference country is defined for which the health costs are determined in the following by utilising the procedure illustrated in Figure 1 and Figure 2.

Then, relevant diseases through food are selected based on Afshin et al. [29] (pp. 1958–1972). The most common diseases caused by malnutrition are cardiovascular diseases (CVD), type 2 diabetes mellitus (T2DM), and neoplasms. For these diseases, the next step is to ascertain the disability-adjusted life years (DALY) for the reference country in general and specific dietary risk factors (r). The DALY measure is often used to quantify the burden of disease on life. It is assumed that every person is born with a certain number of healthy years of life. This number is reduced by disease or premature death, which is reflected in the DALY measure, with the former factored in at a lower rate than the latter. Thus, DALYs are the sum of years lived with disability (YLD) and years of life lost due to premature death (YLL) [33] (pp. 565–569).

From the general DALYs of the diseases (DALY_d_) and those for specific dietary risk factors (DALY_r,d_), the DALY rate (q) of the individual diseases (d) is calculated, by which the percentage of the respective disease burden caused by the individual dietary risk factors is obtained.

q_r,d_ = DALY_r,d_/DALY_d_, (1)

Then, using the COI approach already mentioned, the direct (c_dir_) and indirect costs (c_ind_) of the selected diseases are determined for a given year (y) and the values are summed up. Here, one can distinguish between three cases concerning the data situation.

In the first case, data on both types of costs are available for the same year. Therefore, both values can be added up.

C_d_ = c_dir,d_ + c_ind,d_, (2)

In the second case, data on both types of costs are available for different years. Here, an adjustment is needed with regard to inflation and population size in the summation. This requires information on consumer price indices (CPI_y_) and on population figures (p_y_). In the following calculation, it is assumed as an example that the data on direct costs originate from the year 2010 and the data on indirect costs from the year 2011. Thereby, the costs are standardised to the year 2010.

C_d,2010_ = c_dir,d,2010_ + c_ind,d,cap,2011_ × (CPI_2010_/CPI_2011_) × p_2010_, (3)

In the third case, data is available on both cost types, with one cost type being calculated from the ratio of total costs to per capita costs of the other cost type. In the following calculation, for example, it is assumed that both the direct (c_dir,cap_) and indirect per capita costs (c_ind,cap_) exist, but only the total direct costs (c_dir_) for one year are available.

C_d_ = c_dir,d_ + c_ind,d,cap_ × (c_dir,d_/c_dir,d,cap_), (4)

The next step is to determine the per capita costs for the selected year by dividing the total costs by the population of the specific year. This value is then multiplied by the average inflation rate, calculated from the consumer price indices of the respective years, which gives the inflation-adjusted per capita costs of the selected diseases for the current year.

C_d,cap,y2_ = (C_d,y1_/p_y1_) × (CPI_y2_/CPI_y1_), (5)

In order to ascertain the per capita health costs of the individual dietary risk factors (HC_r,cap,y2_), the inflation-adjusted per capita costs of the selected diseases are first multiplied by the calculated DALY rates, resulting in the per capita health costs of the respective diseases of the individual dietary risk factors (HC_r,d,cap,y2_).

HC_r,d,cap,y2_ = C_d,cap,y2_ × q_r,d_, (6)

The next step is to add up the disease-specific costs calculated for each dietary risk factor.

HC_r,cap,y2_ = ∑_d_ (HC_r,d,cap,y2_), (7)

To get an overview of the total health costs in the reference country, we multiply the differentiated or the summed per capita costs of the dietary risk factors by the population size of the current year.

HC_r,d,y2_ = HC_r,d,cap,y2_ × p_y2_, (8)

HC_r,y2_ = HC_r,cap,y2_ × p_y2_ = ∑_cap_ (HC_r,cap,y2_), (9)

As this illustration is not necessary for further calculation, it is not included in Figure 1.

In order to be able to calculate the quantity-standardised health costs of the harmful quantity that is consumed deficiently or excessively of the respective dietary risk factors on average in the reference country (HHC_r,y2_), one needs the average daily intake of the respective dietary risk factors in the reference country (I_r,cap_) and their daily recommended intake (I^rec^_r,cap_). The former is retrieved from a global database listing the intakes of all dietary risk factors [34]. The latter is obtained either through recommendations of the World Health Organisation (WHO), the German Nutrition Society (DGE), or other official institutions or renowned studies. It can also be determined via the PHD already mentioned. In this guideline, specific intake amounts are given for individual foods. These recommendations are used as a priority in this paper, since the objectives of the health premiums calculated in the end coincide with or complement those of the PHD.

Now we detect the specific harmful amount of the dietary risk factors that is consumed too little or too much per capita daily (x_r,cap_) by calculating the difference between the values just determined.

x_r,cap_ = I_r,cap_ − I^rec^_r,cap_, (10)

Dividing the per capita health costs of each dietary risk factor by the harmful yearly quantity, the quantity-standardised health costs of the harmful quantity are obtained for each dietary risk factor.

HHC_r,y2_ = HC_r,cap,y2_/(x_r,cap_ × 365), (11)

The second part of the method requires a case distinction between dietary risk factors as a single ingredient, whereby in this paper an exclusively excessive consumption is presumed, or as a product category.

In the first case, for the individual ingredients, one needs a reference number of kilocalories consumed daily (a^ref^_cal,day_), which is estimated by the PHD at 2500 kcal per day [16] (pp. 447–492). In addition, information about the number of kilocalories of the product that contains the ingredient is needed (a_p,cal_). From these values, the quantity of the product that would be required if one were to cover the daily requirement exclusively with it is calculated (q_cal_).

q_cal_ = a^ref^_cal,day_/a_p,cal_, (12)

This is then multiplied with the amount of the dietary risk factor contained in the product (m_r,p_) to obtain the total amount of the dietary risk factor that would arise when the daily requirement is completely covered with the product (m_r,day_).

m_r,day_ = q_cal_ × m_r,p_, (13)

Subsequently, the daily recommended intake of the respective dietary risk factors already determined is needed, and is subtracted from the total amount of the dietary risk factor when the daily requirement is completely covered with the product. This shows the harmful amount contained in the product when the daily requirement is fully covered by it (Hm_r,day_).

Hm_r,day_ = m_r,day_ − I^rec^_r,cap_, (14)

The average consumption of the product group in the reference country per day is then determined and the kilocalories of the specific product standardised on this per day is calculated (a_pg,cal,day_). This value is now divided by the reference number of kilocalories consumed daily, which yields the average share of the product in daily consumption in the reference country (q_p,day_).

q_p,day_ = a_pg,cal,day_/a^ref^_cal,day_, (15)

Then, we calculate the average unhealthy amount of the dietary risk factor of the product in the daily consumption in the reference country (n_r,day_) by multiplying the harmful amount of the dietary risk factor when the daily requirement is fully covered by the product with the share just determined. This value indicates how many grams of the dietary risk factor one would consume excessively on average per day in the reference country if one were to rely exclusively on this product within the product group. Although the standardisation is not needed to arrive at the same final result, this intermediate step is included in the method for better and easier understanding.

n_r,day_ = Hm_r,day_ × q_p,day_, (16)

This is then adjusted to the quantity of the product (n_r,p_) by dividing the number of kilocalories of the product by the average consumption of the product group in the reference country per day and multiplying it by the quantity just calculated.

n_r,p_ = n_r,day_ × (a_p,cal_/a_pg,cal,day_), (17)

The last step is to calculate the specific health premium for the product (HP_p_) by multiplying the amount of the dietary risk factor of the product in daily consumption in the reference country by the quantity-standardised health costs of the harmful amount of the dietary risk factor.

HP_p_ = HHC_r,y2_ × n_r,p_, (18)

In the second case, we look at product categories. Here, we consider red meat, processed meat, whole grains, legumes, and fruits. The first step requires the already determined concrete harmful quantity that is consumed deficiently or excessively per capita daily. This harmful amount is calculated from the difference between the actual amount taken in per capita per day and the recommended amount. The underlying data and recommendations are explained in more detail in the following subchapter. It is then necessary to differentiate between dietary risk factors that are consumed too little and those that are consumed too much.

In the case of under-consumed product categories, the harmful quantity calculated above is divided by the daily recommended intake of the respective dietary risk factor to obtain the quantity quota, which represents the basis for internalising health costs (l_r_).

l_r_ = −x_r,cap_/I^rec^_r,cap_, (19)

In the case of over-consumption, the harmful quantity calculated above is divided by the average daily intake of the respective dietary risk factor in the reference country to obtain the quantity quota, which represents the basis for internalising health costs (h_r_). Here, the average daily intake is extracted directly from studies by national or supranational authorities or, if this is not possible, from databases. If aggregated values are not available, the average is determined using population statistics. The underlying data is explained in more detail in the following subchapter.

h_r_ = x_r,cap_/l_r,cap_, (20)

Now we determine the specific amount of the product category or dietary risk factor (m_r_). This is used to calculate the quantity of the product that causes external benefits or costs (m_r,i_).

m_r,i_ = m_r_ × i i = l_r_ if x_r,cap_ < 0; i = h_r_ if x_r,cap_ > 0, (21)

In the last step, the specific health premium for the product category (HP_r_) is calculated by multiplying the quantity of the product category that causes external benefits or costs by the quantity-standardised health costs of the harmful quantity for the dietary risk factor.

HP_r_ = HHC_r,y2_ × m_r,i_, (22)

This represents the end of the method for estimating concrete health premiums of individual dietary risk factors. In the first part of the method, the quantity-standardised health costs of the harmful quantity for each dietary risk factor were determined, and based on this, the specific health premiums for the individual dietary risk factors were calculated in the second part of the method. A case distinction was made between individual ingredients, where only excessive consumption was considered, and product categories. The health premium for under-consumed dietary risk factors has a negative sign, while that for over-consumed dietary risk factors has a positive sign.

### 2.3. Input Data for Quantification and Monetisation

Germany was chosen as the reference country for determining the specific health costs in this paper, as it represents a typical Central European country with comparatively high consumption of meat, high life expectancy, and high education.

#### 2.3.1. Input Data to Quantify the Burden of Diseases

The DALY measure is required to measure the burden of disease on life. Now the identification of the globally most common diseases due to nutrition is accomplished. In 2017, 255 million DALYs arose due to dietary risks, of which 207 million were attributable to CVD, 24 million to T2DM, and 20 million to cancers/neoplasms [29] (pp. 1958–1972).

For these three main diet-related diseases, the DALYs in Germany for 2019 in general and the risk factors for diets high in red meat, processed meat, and sodium, and for diets low in whole grains, legumes, and fruits are calculated below using a tool [30]. Table 1 shows the just mentioned DALYs and the already determined DALY rate of the individual dietary risk factors.

#### 2.3.2. Input Data to Monetise the Burden of Diseases

In order to determine the costs of the respective diseases in Germany, the COI approach is applied.

For cardiovascular diseases, a study by the European Heart Network (2017) is utilised, which contains the direct as well as the indirect expenses for the year 2015 [22]. Here, direct or healthcare costs occur due to primary care, outpatient care, accident and emergency (A and E), inpatient care and medications. The indirect or non-healthcare costs consist of productivity losses due to mortality and morbidity, as well as informal care. Informal care costs represent the opportunity costs of unpaid care and, therefore, measure the amount of money foregone by providing unpaid care [22]. Since these categories of indirect costs are identical for all diseases [22,27,28], renewed explanations are omitted below for simplicity. This results in approximately EUR 28.3 billion in direct costs and EUR 29.2 billion in indirect costs annually in Germany. Germany has the highest healthcare expenses due to CVD in the European Union (EU). In 2015, a total of EUR 57.5 billion in health costs were incurred in Germany due to CVD [22]. After dividing this value by the population in 2015 [35], one gets the per capita costs for that year. These are adjusted for inflation to the year 2022 using an inflation calculator [36], yielding a per capita cost of CVD of EUR 790.78. Table 2 lists the various costs of CVD and the required calculation components.

Numerous studies exist for T2DM, all of which include direct costs. A distinction is made between overall, diabetes-specific, and diabetes-associated direct costs. According to Jacobs et al. (2017) [26] (pp. 855–861), direct expenses of T2DM are incurred by hospitals, pharmacies, physicians, dentists, sick benefits, and other reasons. This resulted in a total of EUR 5146 per capita cost for T2DM patients in 2010, which corresponded to EUR 16.10 billion in total expenditure. Only one study includes indirect costs in addition to direct costs [27]. The indirect per capita costs for people with T2DM are estimated at EUR 4103 for the year 2011 [25]. To make those comparable with direct costs in the next step, they are adjusted for inflation to 2010 using the consumer price indices for 2010 and 2011 [37]. This results in indirect per capita costs for people with T2DM of EUR 4017 in 2010. Multiplying this value by the ratio of total direct costs to per capita direct costs yields the total indirect costs of T2DM of approximately EUR 12.57 billion. In total, therefore, costs of about EUR 28.67 billion for T2DM arise in 2010. Analogous to the procedure for CVD, we divide this value by the population of 2010, and adjust it by an inflationary factor. Finally, we obtain per capita costs for T2DM for the year 2022 at EUR 427.81. Table 3 lists the various costs for T2DM and the required calculation components.

For neoplasms, statistics on cancer from 2018 are used. Although neoplasms are not synonymous with cancer, DALYs caused by them only occur with certain types of cancer [30]. In the case of direct costs, only the share of the costs of cancer drugs in the total health expenditure on cancer care is examined without making any further differentiations. This results in approximately EUR 25.54 billion in direct costs and EUR 21.03 billion in indirect costs. Here, informal care was counted among the indirect costs, whereas in the study it was added neither to the direct nor to the indirect costs [28] (pp. 41–49). The per capita costs of neoplasms for the year 2022 amount to EUR 616.97. Table 4 lists the various costs for neoplasms and required calculation components.

To calculate the total costs of diseases, the most recent population figure for Germany measured on 1 January 2022 of 83,756,658 is applied [38]. This value is made comparable by determining the consumption expenditure of private households for food in Germany for 2021, which amounts to EUR 178.91 billion [39]. If one adjusts this value for the current population and inflation, one arrives at EUR 192.93 billion for the year 2022.

#### 2.3.3. Input Data to Determine Harmful Daily Intakes

Prioritisation is required to determine the average daily intake of the respective dietary risk factor in Germany.

Ideally, studies on the intake of individual dietary risk factors in Germany by German authorities are employed. In the case of daily sodium intake, for example, this paper refers to a study by the Robert Koch-Institute [40], in which daily sodium intake is estimated at an average of 4.10 g.

If such studies are not available, studies by supranational authorities are applied. For daily whole grain intake, for example, a dataset from the European Commission is used, in which the daily intake of German men is estimated at 127.40 g and of German women at 132.60 g [41]. To obtain the average intake in Germany, the quantities are multiplied by the respective population share in Germany, which is shown in Table 5, and one thus arrives at approximately 130.03 g per capita per day.

In the absence of information from intranational or supranational authorities, this paper uses a database from the Gerald J. and Dorothy R. Friedman School of Nutrition Science and Policy (2019), which includes estimated daily intakes of all dietary risk factors for any country [34]. In Germany, an average of 69.10 g of red meat, 35.53 g of processed meat, 10.20 g of legumes, and 169.90 g of fruits are consumed per capita every day.

Different sources are also required to determine the daily recommended per capita intake of the respective dietary risk factors.

For sodium, the suggestion of the German Nutrition Society (DGE) is utilised. It recommends a daily intake of 6.00 g of salt, which corresponds to 2.30 g to 2.40 g of sodium [42] (pp. 62–70).

As there are no recommendations from the authorities or the PHD regarding the intake of processed meat, a suggestion by Afshin et al. (2019) is used [29] (pp. 1958–1972). They propose an per capita intake of 2.00 g of processed meat per day, ranging from 0.00 g to 4.00 g. Another alternative would be to assume that all processed meat consumed is harmful, resulting in an optimal daily amount of 0.00 g per capita [43]. In this paper, it is expected that a strong reduction in the first step is more effective than to completely renunciate. The goal of a total renunciation can be set in the next step if the reduction is carried out successfully, but it is unrealistic in the beginning. For red meat, whole grains, legumes, and fruits, the scientific targets for a planetary health diet are used. The optimal daily intake amounts to 14.00 g of red meat, 232.00 g of whole grains, 75.00 g of legumes, and 200.00 g of fruits [16] (pp. 447–492).

#### 2.3.4. Input Data to Calculate Concrete Health Premiums for Products

Table 6 shows the average and recommended daily per capita intake amounts of the just described dietary risk factors in Germany. Furthermore, the concrete harmful quantities (x_r,cap_) that are consumed deficiently or excessively of the dietary risk factors per capita daily and the quantity quota which represents the basis for internalising health costs (l_r_/h_r_) are calculated there.

The just mentioned quantity quota is required when calculating the health premiums for dietary risk factors that are available as product groups. In addition, there is a necessity for a concrete quantity for which external health costs are internalised. In this paper, in the first step, premiums and discounts are calculated for one kilogram of the respective dietary risk factor. In the second step, this is adjusted to the quantities of selected foods. Furthermore, information on the prices of the specific products is required. This study examines some products of Edeka, a large German retailer. Here, apples [44], minced meat [45,46], salami [47,48], wholemeal bread [49], and legumes [50] are assessed. In addition, more information is obtained via a self-created database.

To calculate the health premiums of dietary risk factors as individual ingredients, the daily consumed reference number of kilocalories already addressed in Section 2.2 is needed, which is estimated by Willett et al. (2019) to be 2500.00 kcal [16] (pp. 447–492). Since sodium is the only single ingredient considered, we refer to it exclusively in the following.

In this paper, the health premiums due to sodium are calculated for four products as examples. For these products, one needs the selling price, the specific number of kilocalories, and the amount of sodium contained. Here, we determine corresponding health premiums for the types of salami from Edeka already mentioned above and for gouda [51], as well as mozzarella [52] from Penny-market, another large German retailer. This is accomplished by determining values on the average daily per capita consumption of the product groups cheese and salami. For cheese, this amounts to 69.32 g, according to the latest statistics from the Federal Ministry of Food and Agriculture in Germany [53]. On average, 72.05 g of salami is consumed daily per capita in Germany [54].

Finally, the health costs for certain food products from the Penny-market are determined and applied to a study that already calculated the influence of external environmental costs on corresponding products [55]. The values originate from the year 2020 and are accordingly adjusted to the current year with an inflation factor. In this paper, we assess gouda, mozzarella, apples, mixed minced meat, and bananas, and calculate the corresponding true prices resulting from internalising external environmental and health costs. Here, already calculated health premiums of gouda and mozzarella are applied uniformly and independently of the production method to organic and conventional prices, as their nutritional composition does not differ significantly [56].

## 3. Results

**Figure 3:**
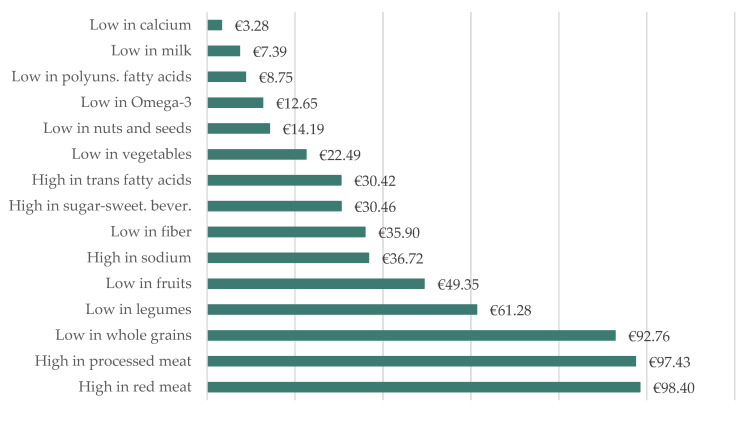
Per capita costs of dietary risk factors in Germany in 2022.

Results show that EUR 601.48 per capita health costs occur annually in Germany due to dietary risk factors. The six largest dietary risk factors account for 72.48% of the costs, which is why we focus on these factors in this paper. Figure 3 lists the health costs of the individual dietary risk factors. The highest per capita costs are incurred by diets high in red meat with EUR 98.40 (16.36% of total costs), followed by diets high in processed meat with EUR 97.43 (16.20% of total costs). Over-consumption of red meat is responsible for 452,267.18 DALYs and over-consumption of processed meat for 366,455.26 DALYs per year. This is followed by diets low in whole grains with EUR 92.76 (15.42% of total costs) and 543,258.26 DALYs, diets low in legumes with EUR 61.28 (10.19% of total costs) and 397,559.75 DALYs, as well as diets low in fruits with EUR 49.35 (8.20% of total costs) and 253,199.84 DALYs per year. The sixth highest costs are incurred by diets high in sodium with EUR 36.72 (6.11% of total costs) and corresponding 242,073.00 DALYs. Overall, EUR 293.42 per capita health costs (48.78% of total costs) and 1,365,831.01 DALYs arise annually due to over-consumption of food and EUR 308.05 per capita health costs (51.22% of total costs) and 1,823,748.19 DALYs due to under-consumption of food in Germany.

**Figure 4:**
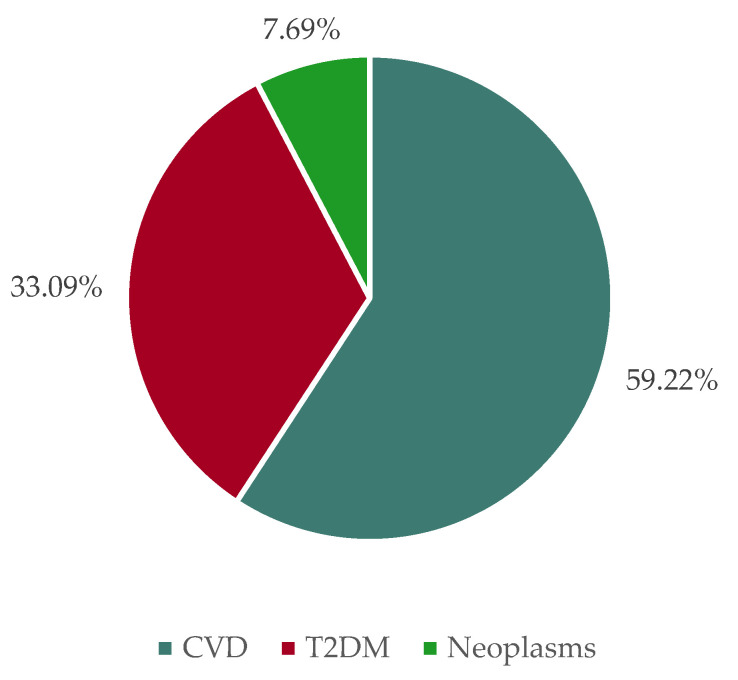
Shares of cardiovascular diseases, type 2 diabetes mellitus, and neoplasms in total health costs due to food consumption in Germany in 2022.

The total annual external health expenses of CVD, T2DM, and neoplasms caused by dietary risk factors are approximately EUR 50.38 billion based on the most recent population status as of 1 January 2022. Figure 4 shows the distribution of health costs among these three diseases. Here it becomes apparent that of the total costs, EUR 29.84 billion (59.22% of total costs) are caused by CVD, EUR 16.67 billion (33.09% of total costs) by T2DM and EUR 3.87 billion (7.69% of total costs) by neoplasms. If one compares these total annual external costs with annual private household consumption expenditure on food of EUR 192.63 billion in Germany [39], one finds that slightly more than a quarter of this corresponds to the value of the costs incurred due to health externalities.

**Figure 5:**
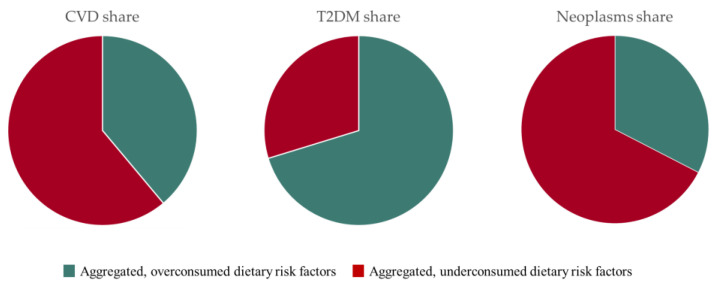
Share of health costs due to under- and over-consumption of food in the respective disease in Germany in 2022.

Over-consumption occurs when the amount that is actually consumed exceeds the recommended amount, while under-consumption refers to the opposite. Table 6, included in the previous chapter, shows the data required for this. The following explanations are visualised in Figure 5. Here, the shares of over- and under-consumption in the three main diseases caused by malnutrition are shown.

Of CVD health costs from dietary risk factors, 38.89% are the consequence of over-consumption, while 61.11% are attributable to under-consumption. Legumes with 17.20% and whole grains with 17.07% account for the highest share of the resulting total CVD health expenses. Red meat and sodium turn out to cause the largest shares of health costs due to over-consumption with 11.32% and 9.80%, respectively.

T2DM occurs mainly because of excessive consumption of dietary risk factors. Of the health costs, 70.27% are due to over-consumption, of which 84.22% are ascribable to red and processed meat. Excessive consumption of the two types of meat thus poses the greatest risk for dietary T2DM. The remaining 29.73%, which occur due to undersupply, are mainly caused by deficient consumption of whole grains and fruits.

Neoplasms result predominantly from an undersupply in the diet. Thus, 67.44% of the health costs are attributable to under-consumption, of which 37.02% are traced back to a too low daily intake of whole grains. The remaining 32.56% largely arise from red and processed meat. Excessive consumption of the two types of meat is responsible for 87.98% of the health costs from neoplasms due to over-consumption.

In summary, CVD and neoplasms are predominantly caused by an undersupply of food. In both cases, too little consumption of whole grains is elementary. T2DM is mainly a consequence of over-consumption of food, with red and processed meat being the main contributors. Red and processed meat also contribute largely to the health costs of neoplasms and especially red meat to the health costs of CVD.

Table 7 depicts that the quantity-standardised health costs of the harmful quantity, which represent the basis to incentivise consumption reduction, amount to 0.49 cents (ct) per gram for red meat, 0.80 ct per gram for processed meat, and 5.59 ct per gram for sodium. The cost of the harmful quantity that represents the basis in terms of incentives for higher consumption is 0.25 ct per gram for whole grains, 0.26 ct per gram for legumes, and 0.44 ct per gram for fruits.

For dietary risk factors as whole product groups, we calculate health premiums for each kilogram, which are presented in Table 8. This amounts to EUR 3.90 for red meat and EUR 7.51 for processed meat. For under-consumed product groups, negative values result. The health discount for whole grains is EUR 1.10 per kilogram, EUR 2.24 per kilogram for legumes, and EUR 0.68 per kilogram for fruits.

**Figure 6:**
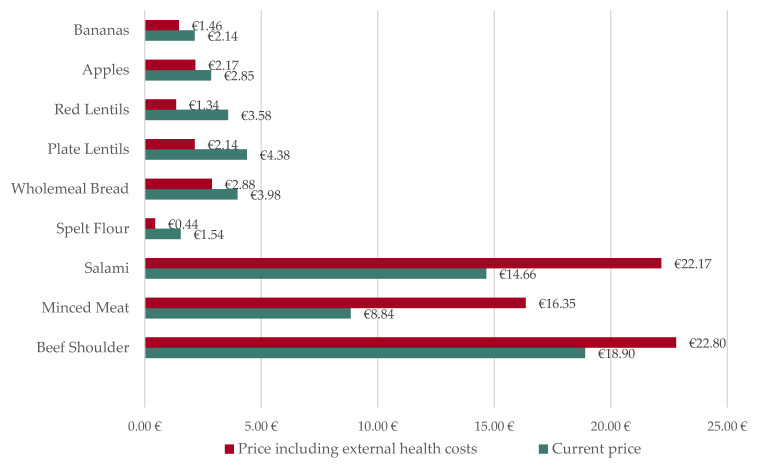
Price changes of certain Edeka products with the internalisation of health costs into their prices in Germany in 2022.

To illustrate the concrete impact of internalised health costs on food, we ascertain the price changes of selected products from Edeka, one of the leading retailers in Germany, in Figure 6. A quantity of one kilogram is always considered. The price of beef shoulder rises from EUR 18.90 to 22.80 (price increase of 20.64%), the price of minced meat expands from EUR 8.84 to 16.35 (price increase of 84.98%), and the price of salami increases from EUR 14.66 to 22.17 (price increase of 51.25%). If the health expenses of wholemeal products are internalised, the price of spelt flour falls from EUR 1.54 to 0.44 (price reduction of 71.13%) and the price of wholemeal bread from EUR 3.98 to 2.88 (price reduction of 27.52%). When the health costs of legumes are priced in, the price of plate lentils declines from EUR 4.38 to 2.14 (price reduction of 51.11%) and the price of red lentils from EUR 3.58 to 1.34 (price reduction of 62.53%). Internalising the health costs of fruits, the price of apples decreases from EUR 2.85 to 2.17 (price reduction of 23.76%) and the price of bananas drops from EUR 2.14 to 1.46 (price reduction of 31.59%).

With the information on the quantity-standardised health costs of sodium, the average daily reference number of kilocalories and the data on individual products, their specific health premiums are obtained. Table 9 shows that the health costs for the 100 g pack of organic salami from Edeka are 8.14 ct (price increase of 4.82%). For 100 g of Edeka’s conventional salami, the health cost premium amounts to 7.17 ct (price increase of 5.79%). Gouda from Penny-market would become 2.41 ct more expensive with internalised health costs of sodium (price increase of 1.05%). Penny-market’s 220 g mozzarella generates external health costs of 0.13 ct (price increase of 0.23%).

The inflation-adjusted external ecological cost premiums of certain products from the above-mentioned Penny-market in some cases differ greatly depending on the production practice. Thus, the organic and conventional variants of the same product entail different environmental cost premiums. Since the production method has no significant influence on the extent of external health expenses, the cost premiums and discounts for the organic and conventional variants of the product are assumed to be identical. In the following, one kilogram is always applied as the reference quantity. The environmental surcharge for conventional gouda is EUR 4.69, while that for organic gouda is EUR 3.49. For both, the health expenses amount to EUR 0.16. For conventional mozzarella, the environmental cost premium is EUR 3.04, and for organic mozzarella EUR 2.29. The health costs of both variants come to EUR 0.01. Conventional mixed minced meat should become more expensive by EUR 10.35, and organic mixed minced meat by EUR 12.39 with the internalisation of external environmental costs. The health cost surcharge is EUR 7.51. Conventional apples should become higher priced by EUR 0.19, and organic apples by EUR 0.13 when internalising environmental costs. For conventional bananas, the environmental surcharge is EUR 0.21, and for organic bananas EUR 0.16. If health costs are internalised, both fruits just mentioned become cheaper by EUR 0.68, which corresponds to overcompensation of ecological costs.

**Figure 7:**
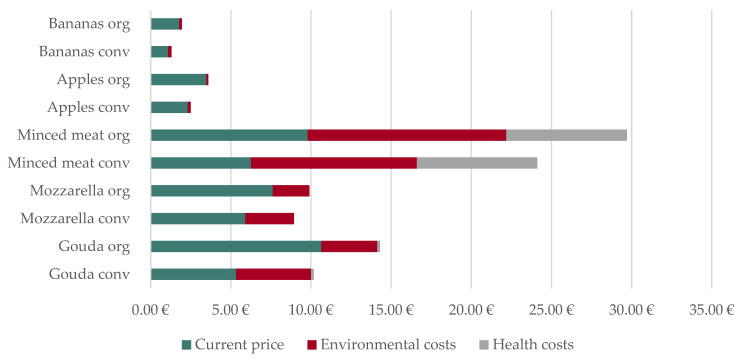
Current prices of food from the Penny-market and their environmental and health costs in Germany in 2022.

Figure 7 illustrates that the simultaneous internalisation of external environmental and health costs leads to the greatest price change for mixed minced meat. Thus, the price of conventional minced meat would increase from EUR 6.26 to 24.12 per kilogram (385.32% of the current price), while that of the organic variant would rise from EUR 9.80 to 29.70 (303.07% of the current price). For gouda and mozzarella, the percentage price expansion of the conventional variant is also greater than that of the organic variant. The price of conventional gouda would climb from EUR 5.33 to 10.18 (190.96% of the current price), and that of organic gouda from EUR 10.65 to 14.30 per kilogram (134.27% of the current price). A kilogram of conventional mozzarella would be priced EUR 8.95 instead of EUR 5.91 (151.55% of the current price), and a kilogram of organic mozzarella would be priced EUR 9.91 instead of EUR 7.62 (130.14% of the current price).

This effect can also be observed with price reductions. Here, too, the percentage price decrease is greater for the conventional variants than for the organic variants. Thus, the price of conventional apples would drop from EUR 2.31 to 1.83 per kilogram (79.08% of the current price), while that of the organic variety would decline from EUR 3.47 to 2.92 (84.20% of the current price). The price per kilogram of conventional bananas would diminish from EUR 1.09 to 0.63 (57.67% of the current price), and the price per kilogram of organic bananas would decrease from EUR 1.79 to 1.27 (71.15% of the current price).

## 4. Discussion

Now that the external health costs of food in Germany resulting from nutrition have been presented and the impact of internalising these expenses on consumer prices has been evaluated, the results and the uncertainties will be discussed.

First, we describe, explain, and discuss the dealing with uncertainties based on the input data breakdown in Section 2.3, then we discuss our results, and finally, we briefly address various implementation options and their feasibility.

### 4.1. Dealing with Uncertainties

This paper uses a variety of data from different sources, each of which is subject to uncertainty. In measuring the burden of disease on life, a tool from the University of Washington is employed [30]. This provides an overview of the DALYs for the year 2019, whereby the input data can be varied flexibly. In this context, the DALYs represent the mean value of a range, which in some cases is considerable. As there is no sensitivity analysis in this study, the DALY estimates are subject to some uncertainty. Also, the limitation of the three diseases CVD, T2DM, and neoplasms excludes the occurrence of other diseases by dietary risk factors. Thereby, other diseases also represent a part of the appearing DALYs, but their costs are not calculated, which is why they illustrate an externality even after applying the method. However, it can be assumed that these diseases would have a comparatively small influence on the results as they only contribute 0.79% of the DALYs caused by nutrition. Moreover, if one adds up the individual DALYs of the total dietary risk factors for the respective diseases, one gets higher values than those of the tool, which are on average, related to CVD, T2DM, and neoplasms, about 22,74% lower than the sum over the DALYs of all dietary risk factors. The reason for this is interdependencies between the individual dietary risk factors. Dietary fiber, for example, is found in fruits, vegetables, whole grains, and legumes [57] (pp. 411–418), and a delimitation is, therefore, not possible. The same applies to sodium, which is found in red and processed meat, for example, and is, hence, already included in their DALYs. Especially with the just mentioned meat varieties, a clear demarcation is impossible, as a certain amount of red meat is processed. These interdependencies result in an overestimation of a part of the costs calculated in this study. The DALYs of dietary risk factors due to CVD is overestimated by a factor of about 1.40, due to T2DM by about 1.20 and to neoplasms by about 1.30. For salami, for example, both health costs resulting from excessive consumption of processed meat and those resulting from excessive consumption of sodium were calculated. Since it can be assumed that the former already includes the latter, internalising both costs in the price of the same product is not appropriate.

In determining the costs of the selected diseases, aggregated costs for the super categories of diseases are used in this study. Concerning CVD, there is no subdivision into individual subcategories such as ischemic heart diseases or stroke. In neoplasms, the different types of cancers are not differentiated. As a result, the costs of illness cannot be calculated in a fully differentiated way for each dietary risk factor. Because of the coarser granulation, the total costs are higher than the sum of the individual costs in a more disaggregated view. This problem is counteracted in this study by using the DALY rate, as this normalises the costs to the over category under consideration.

Furthermore, significant uncertainties arising from the heterogeneous sources regarding the age of the data and the data contained exist, which is particularly important when calculating the costs of diabetes. Numerous studies are published containing varying direct costs, most of which are more than ten years old. In this paper, the study with the highest sample size was selected, as it is the most representative. It represents almost all people with T2DM in Germany, and its result is similar to that of the Costs of Diabetes Mellitus (CoDiM) study, which analysed data from the same year from about 1.5 million insured people and came up with a per capita cost of EUR 5239 [23] (pp. 510–516). Furthermore, it can be assumed that other studies that estimate the direct per capita costs of people with T2DM at EUR 4377 for 2010 [24] (pp. 951–957) and EUR 3352 for 2011 [25] are less representative than the study mentioned at the beginning due to their sample size. Only one study includes indirect costs from 2011, which probably underestimates them. Hence, there is no comparative value to evaluate the data of this source.

One of the key uncertainties in this paper is the application of different recommended and actual intakes of dietary risk factors. Thus, there are some inconsistencies in the quantities actually consumed in Germany. If one compares the daily intake of whole grains in Germany by the EU Commission source to that of the global database, for example, different values are available. This is mainly attributable to the year in which the data was collected, as older datasets do not include the current changes in eating habits in society. Since the former source is more recent, this value is probably more representative. In our study, both sources are used synchronously, because although some inconsistencies occur, most of them are minor and the majority of the daily intake amounts can only be determined via the database.

Furthermore, for four out of six of the dietary risk factors used here as examples for calculation, the daily per capita reference quantity is ascertained based on the PHD. The PHD provides revenue values that have been calculated by a scientific commission but are not yet represented in any guideline. Besides, the possible range of the individual macronutrient intake recommendations described in this study is very large.

For sodium, the WHO recommends a maximum of 2.00 g per day [58], which is supported by the European Food Safety Authority [59]. In contrast, the United States Department of Agriculture (USDA) recommends a maximum daily intake of 2.30 g of sodium [60]. The German Nutrition Society (DGE) suggests a similarly high intake of 6.00 g of salt per day, which corresponds to 2.30 g to 2.40 g of sodium [42] (pp. 62–70). Although the WHO endorses a maximum daily intake of 5.00 g of salt or 2.00 g of sodium in the course of a strong recommendation, the proposal of the German Nutrition Society (DGE) of 2.30 g of sodium per day is applied for Germany in this paper, as this is in line with the WHO’s goal of reducing the global intake of salt in the population by 30.00% [42,61].

Another study fundamentally questions this suggestion. The authors assume that a consumption of 3.00 g to 5.00 g of sodium per day is associated with the lowest risk of cardiovascular diseases. The authors, therefore, endorse a maximum reference amount of 5 g of sodium per day [62]. Utilising the DGE suggestion in this paper is a sensible choice due to the consistency with the WHO’s goals and the comparability with other advice, but it is subject to fundamental uncertainties regarding the optimal and consequently harmful amount of sodium intake.

The recommended daily intake of processed meat is assumed to be 2.00 g, which is proposed by Afshin et al. (2019), assignable to the lack of national and international guidelines [29] (pp. 1958–1972). This value underlies a certain degree of uncertainty as it is subject to a wide range and a lack of justification.

In addition, the already mentioned University of Washington tool contains some difficulties regarding the terms defined. Thus, the harmful quantities determined in that tool, by which corresponding DALYs occur, differ slightly in some cases, but also strongly in others, from those used in this paper. For red meat, for instance, a maximum per capita reference amount of four ounces per week is suggested, which corresponds to about 16 g per day. Compared to the recommended PHD intake of 14.00 g per day, the deviation is not strong. For whole grains, however, the deviation is significant. Diets low in whole grains are defined as such in this tool if less than 113.40 g per capita is consumed daily [43]. In contrast, this paper assumes that a daily whole grain consumption of less than 232.00 g per capita is not optimal.

To calculate the health costs of the Penny-market products, prices from the year 2020 are used to enable comparability with the study mentioned. Although these are adjusted to current inflation in this paper, no external price increases are included, which is why slight distortions may occur.

### 4.2. Discussion of Results

**Figure 8:**
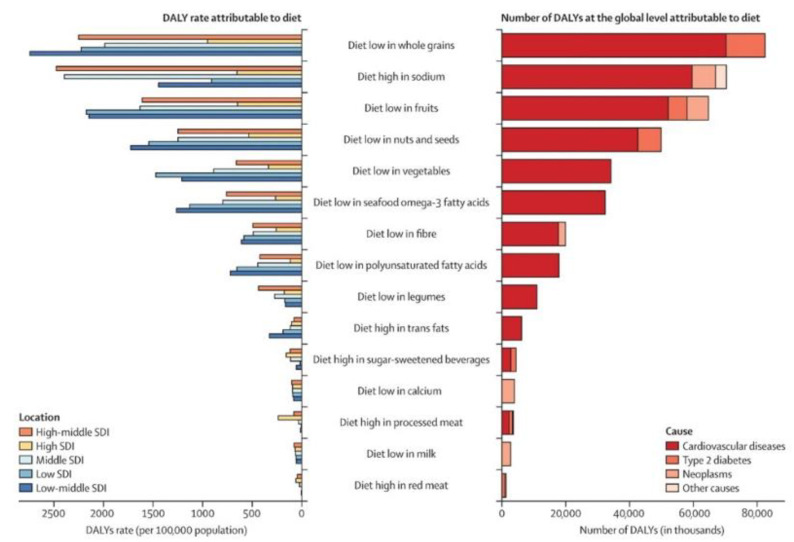
Number of deaths and DALYs, and age-standardised mortality rate and DALY rate (per 100,000 population) attributable to individual dietary risks at the global and SDI levels in 2017. Source: Afshin et al. (2019) [29] (pp. 1958–1972).

Comparing the most influential dietary risk factors in Germany with the most influential globally, illustrated in Figure 8, it becomes apparent that in both cases, most DALYs are caused by diets low in whole grains. While on a global average one loses comparatively few years of life through diets high in red and processed meat, this is reversed for Germany. Thus, over-consumption of red meat leads to the second highest DALYs, and over-consumption of processed meat to the fourth highest DALYs in Germany. If these two values are aggregated into a total value, it becomes evident that in Germany, in terms of nutrition, the healthiest years of life are lost annually through meat consumption. The reason for the enormous deviation of the average global values from those for Germany is the comparatively high annual consumption of meat in Germany, which is about 76.85% higher than in the global average [63]. While diets high in sodium and diets low in fruits are among the most influential risk factors from both a global and a German perspective, the negative health effect of diets low in legumes in Germany is significantly higher than the international reference value.

The number of DALYs of the individual diseases is positively correlated with the resulting per capita health costs. This means that a higher number of DALYs from CVD, for example, also leads to more health costs. However, a supposed inconsistency in this pattern can be observed in the aggregated DALYs of the individual dietary risk factors across the different diseases. In Germany, for example, the healthiest life years are lost through an undersupply of whole grains, but the resulting health costs are lower than those of diets high in red and processed meat. The reason for this is the underlying shares of the DALYs of the dietary risk factors in the total DALYs of the respective diseases in Germany, expressed by the DALY rate. It is important to understand that the same number of DALYs for T2DM of a dietary risk factor carries significantly more weight than for CVD and neoplasms. Differences in the level of total DALYs caused by the three diseases in Germany constitute the missing explanation. While this is at a relatively equal level for CVD and neoplasms, it is more than four million DALYs lower for T2DM than for the other two. Since over-consumption of the two types of meat accounts for more than a quarter of the DALYs due to T2DM, the corresponding share in health costs of the disease is relatively high.

CVD and neoplasms in Germany are mainly caused by undernutrition. This finding is in line with global values. For neoplasms, the corresponding global cause shares are almost identical to those in Germany. Regarding CVD, the global share of DALYs assignable to undernourishment is 18.59% higher than in Germany. A significant difference arises in the case of T2DM. While 70.27% of life years lost annually in Germany are caused by excessive consumption of dietary risk factors, 87.42% of the global DALYs of T2DM arise as a result of not eating sufficiently [29] (pp. 1958–1972). This serious difference mainly exists due to the combination of a high Socio-demographic Index (SDI) and a resulting comparatively very high consumption of meat in Germany. The SDI represents an average of the rankings of per capita income, average education level and fertility rate for individual countries [64]. Globally, developmental undernutrition plays an important role. It is assumed that early-life undernutrition in combination with later obesity leads to a higher risk of developing T2DM [65] (pp. 1773–1778). The finding that high consumption of red and processed meat in particular leads to T2DM must be critically questioned. For example, Sanders et al. (2022) [66] found no correlation between red meat consumption and an increase in most glycaemic and insulinemic risk factors for T2DM. This was compared to diets with reduced or no red meat. Further research is needed to determine a possible link between high red meat consumption and T2DM.

In comparison with calculated environmental food price premiums, no distinction is made between different production methods when determining health costs. If a price for both the organic and the conventional variant is available, the average of these is used. This is permissible because, according to Smith-Spangler et al. (2012) [56], organic and conventional products differ in their nutritional composition only in phosphorus and phenols and, thus, no significant difference occurs in the resulting health costs. For this reason, the conventional and organic variants of the same product have identical price premiums. This would not be the case if the impact of especially air pollution on human health were taken into account when calculating health costs. Perotti (2020) classifies this nutritional impact as an environmental human health impact, which highlights the difficult distinction between external environmental and health aspects of food consumption [18]. Since the impact of food production on human health due to, for example, reactive nitrogen and energy are already included in the ecological costs calculated by the study [55] used to illustrate an integrated internalisation of external ecological and health costs and since a fully delimited consideration in terms of the impact on human life is impossible, this paper refrains from an investigation. Moreover, other externalities such as health effects of food poisoning, health effects of pesticide exposure, and health effects of antibiotic use subsist [18]. An additional monetisation and a subsequent internalisation of the just mentioned externalities would result in higher health costs for the individual dietary risk factors. These are not taken into account in this paper, as their importance and influence can be estimated at a relatively low level due to the lack of inclusion in other large studies such as that of the scientific group of the U.N. [10].

As already illustrated, the majority of diseases occur due to an undersupply of food. Consequently, there should be an incentive to consume more of it. Based on the calculated results, under-consumed food becomes cheaper when both health costs and environmental costs are internalised, because the impact of health externalities exceeds that of environmental externalities. However, for over-consumed food, both types of costs add up, whereby the share of environmental costs is greater than that of health costs for animal-based and dairy products used in the aforementioned study [55].

The Boston Consulting Group (BCG) estimates the total environmental external costs of agriculture in Germany at around EUR 90.77 billion in 2018 [67]. Adjusted for inflation, this corresponds to approximately EUR 103.48 billion for the year 2022. This means that in Germany, a total of approximately EUR 153.86 billion in external costs are caused by nutrition, of which approximately 67.26% amount to ecological and approximately 32.74% to health aspects. Relatively speaking, both values are within the ranges of the U.N. study. Although the costs to human life in this study are estimated to be higher than the environmental costs, the result of this paper is still plausible for Germany [10] because of the high SDI of Germany. The relatively high per capita income and a high level of education result in high consumption and consequently increased production with a simultaneous awareness of the negative consequences of an unhealthy diet. The chance of undernutrition thus decreases compared to overnutrition resulting in more environmental costs and fewer health costs due to undernourishment. This in turn explains why health costs in Germany account for a substantially smaller share of consumption expenditures by food than in international comparison [10]. Globally, it can be assumed that a decreasing SDI leads to less consumption, less education, and an associated increased risk of undernutrition. One example of why the intake of knowingly harmful foods is still significant in high SDI countries is meat. Although it has been proven that in high-income countries meat consumption decreases with education, income, and social class [68,69], it is a part of important traditions due to its participation in the biological and cultural evolution of humans [70,71]. This also explains why globally, 20.53% of dietary DALYs are caused by an oversupply each year, while in Germany this figure is at 42.82%.

Thus, in comparison, the production increases in Germany, which leads to higher environmental costs, while, because the diseases examined here are in general mainly caused by an undersupply of food, there are fewer external health impacts on human life, explaining the dominance of environmental costs over health costs in Germany.

The results illustrate that an integrated internalisation of all external costs from food consumption leads to incentives for a healthier diet. Food that is taken in deficiently becomes cheaper for the consumer, and food that is taken in excessively becomes more expensive. In the process, poor production methods and the resulting environmental costs or benefits increase or decrease the calculated health costs. Thus, the side effects of economic activity are monetised and become part of prices. The market is equalised, and a shift to healthier and more sustainable diets is motivated.

## 5. Conclusions

In this study, a methodology is developed to determine the health costs of individual foods and their ingredients for the reference country to, thus, create reference values for potential price increases or decreases. In our framework, first, a new type of DALY rate is introduced to determine the percentage of the diseases CVD, T2DM, and neoplasms of the most influential dietary risk factors in their total numbers in Germany. This rate is used in the next step to calculate the per capita health costs of the individual dietary risk factors using the COI approach. By determining harmful levels of dietary risk factors, quantity-standardised health costs are calculated, and concrete health premiums or discounts for individual ingredients as well as individual products are determined.

We find, that EUR 601.50 per capita in total health costs are incurred in Germany per year due to nutrition. Almost one third of these is caused by an over-consumption of meat, thus, highlighting an excessive meat consumption as a main driver of health costs in Germany. Further results underline the insufficient consumption of grains, legumes, and fruits, as well as the excessive consumption of sodium as significant drivers of health costs.

This thoroughly answers the research question formulated in the introduction.

Overall, 48.78% of health costs in Germany are due to an oversupply of food, while 51.22% are caused by an undersupply. While both globally and in Germany most DALYs are incurred by an undersupply of food, the proportion in Germany is significantly lower than in international comparison, which is primarily due to Germany’s high consumption of meat. Comparing the calculated health costs of minced meat, apples, bananas, gouda, and mozzarella to the environmental costs calculated by Michalke et al. (2019) [55], an integrated internalisation of both costs would make over-consumed food more expensive and under-consumed food cheaper, hence, providing incentives for a healthier diet.

In summary, the costs of nutrition through impacts on human life, which to date are not included in market prices and thus not compensated, can be quantified and monetised by applying the methodology developed in this paper. By pricing food appropriately, the 17 SDGs could be advanced by improving the health aspect in particular. For this, an international application in addition to the calculation of health costs through this method for Germany is required for a globally healthier diet and an equalisation of the market.

## Figures and Tables

**Table 1:** DALYs in Germany in 2019 and resulting DALY rates differentiated by disease due to selected dietary risk factors.

		Diseases		
Dietary Risk Factor Diets…		CVD	T2DM	Neoplasms
…high in red Meat	DALYs DALY rate	261,516.67 0.051	120,508.68 0.117	70,241.83 0.013
…high in processed Meat	DALYs DALY rate	158,826.85 0.031	163,747.32 0.159	43,881.09 0.008
…low in whole Grains	DALYs DALY rate	394,528.87 0.077	49,234.42 0.048	99,494.97 0.019
…low in Legumes	DALYs DALY rate	397,559.75 0.077	- -	- -
…low in Fruits	DALYs DALY rate	149,354.58 0.029	47,852.02 0.046	55,993.24 0.011
…high in Sodium	DALYs DALY rate	226,479.22 0.044	- -	15,593.78 0.003
Total (not only because of the Diet)	DALYs DALY rate	5,129,871.3 1	1,032,452.88 1	5,316,908.62 1

The DALY rate of cardiovascular diseases in red meat, for example, is calculated by q_red meat, CVD_ = 261,516.67/5,129,871.30 = 0.051.

**Table 2:** Direct and indirect costs of cardiovascular diseases in 2015, calculation components, and calculated per capita costs in 2015 and 2022 in Germany.

CVD	
**Direct (2015)**	
Primary Care	3,215,531,000.00 €
Outpatient Care	4,730,257,000.00 €
A&E	115,223,000.00 €
Inpatient Care	14,965,129,000.00 €
Medications	5,279,912,000.00 €
C_dir,CVD,2015_	28,306,052,000.00 €
**Indirect (2015)**	
Prod. Loss from prem. Mort.	8,556,221,000.00 €
Prod. Loss from Morbidity	8,495,837,000.00 €
Informal Care	12,149,160,000.00 €
C_ind,CVD,2015_	29,201,218,000.00 €
**Total**	
C_CVD,2015_	57,507,270,000.00 €
y_1_	2015
p_2015_	82,175,684.00
CPI_2022_/ CPI_2015_	1.13
C_CVD,cap,2015_	699.81 €
C_CVD,cap,2022_	790.78 €

**Table 3:** Direct and indirect costs of type 2 diabetes mellitus in 2010 and 2011, calculation components, and calculated per capita costs in 2010 and 2022 in Germany.

T2DM	
**Direct (2010)**	
Hospitals/ Inpatient Treatment	1,986.00 €
Pharmacies/ Medication	1,285.00 €
Physicians/ Outpatient care	786.00 €
Dentists	155.00 €
Sick Benefits	149.00 €
Other	785.00 €
c_dir,T2DM,cap,2010_	5,146.00 €
C_dir,T2DM,2010_	16,100,000,000.00 €
**Indirect (2011)**	
c_ind,T2DM,cap,2011_	4,103.00 €
CPI_2010_/ CPI_2011_	0.98
**Indirect (2010)**	
c_ind,T2DM,cap,2011_	4,016.80 €
C_ind,T2DM,2010_	12,567,143,526.67 €
**Total**	
C_T2DM,2010_	28,667,143,526.67 €
y_1_	2010
p_2010_	81,715,602.00
CPI_2022_/ CPI_2010_	1.22
C_T2DM,cap,2010_	350.66 €
C_T2DM,cap,2022_	427.81 €

**Table 4:** Direct and indirect costs of neoplasms in 2018, calculation components, and calculated per capita costs in 2018 and 2022 in Germany.

Neoplasms	
**Direct (2018)**	
Health Expenditure on Care	25,537,000,000.00 €
C_dir,neoplasms,2018_	25,537,000,000.00 €
**Indirect (2018)**	
Informal Care	5,141,000,000.00 €
Prod. Loss from prem. Mort.	11,516,000,000.00 €
Prod. Loss from Morbidity	4,370,000,000.00 €
C_ind,neoplasms,2018_	21,027,000,000.00 €
**Total**	
C_neoplasms,2018_	46,564,000,000.00 €
y_1_	2018
p_2018_	83,019,213.00
CPI_2022_/ CPI_2018_	1.10
C_neoplasms,cap,2018_	560.88 €
C_neoplasms,cap,2022_	616.97 €

**Table 5:** Population share in Germany in 2021 and 2022.

Population: Germany, Reference Date, Sex			
Reference Date	Sex		
	Male	Female	Total
31 December 2021	41,066,785.00	42,170,339.00	83,237,124.00
	49.34%	50.66%	100.00%
1 January 2022			83,756,658.00

Source: Statistisches Bundesamt (2022) [37].

**Table 6:** Average, recommended, and harmful daily per capita intake amounts of selected dietary risk factors in Germany in 2022, and the calculated quantity quota.

	Dietary Risk Factors					
	Diets High in…			Diets Low in…		
	…Red Meat	…Processed Meat	…Sodium	…Whole Grains	…Legumes	…Fruits
I_r,cap_ [g]	69.10	35.53	4.10	130.03	10.20	169.60
I^rec^_r,cap_ [g]	14.00	2.00	2.30	232.00	75.00	200.00
x_r,cap_ [g]	55.10	33.53	1.80	–101.97	–64.80	–30.40
i (h_r_/l_r_)	0.80	0.94	-	0.44	0.86	0.15

**Table 7:** Harmful daily per capita intake amounts, annual per capita costs, and quantity-standardised health costs of selected dietary risk factors in Germany in 2022.

	Dietary Risk Factors					
	Diets High in…			Diets Low in…		
	…Red Meat	…Processed Meat	…Sodium	…Whole Grains	…Legumes	…Fruits
x_r,cap_^1^ [g]	55.1000	33.5300	1.8000	–101.9700	–64.8000	–30.4000
HC_r,cap,2022 _^2^ [€]	98.3983	97.4259	36.7219	92.7638	61.2849	49.3488
HHC_r,2022 _^3^ [€/g]	0.0049	0.0080	0.0559	–0.0025	–0.0026	–0.0044

^1^ The specific harmful amount of the dietary risk factor that is consumed too little or too much per capita daily; ^2^ Per capita health costs of the individual dietary risk factor; ^3^ Quantity-standardised health costs of the harmful quantity of the individual dietary risk factor

**Table 8:** Calculation components and calculated health premiums and discounts for selected dietary risk factors in Germany in 2022.

	Dietary Risk Factors				
	Diets High in…		Diets Low in…		
	…Red Meat	…Processed Meat	…Whole Grains	…Legumes	…Fruits
HHC_r,2022_^1^ [€/g]	0.0049	0.0080	–0.0025	–0.0026	–0.0044
i (h_r_/l_r_) ^2^	0.7974	0.9437	0.4395	0.8640	0.1520
m_r_^3^ [g]	1000.0000	1000.0000	1000.0000	1000.0000	1000.0000
m_r,i_^4^ [g]	797.3951	943.7095	439.5259	864.0000	152.0000
HP_r_^5^ [€]	3.9014	7.5125	–1.0955	–2.2387	–0.6760

^1^ Quantity-standardised health costs of the harmful quantity of the individual dietary risk factor; ^2^ The quantity quota that represents the basis for internalizing the health costs of overconsumed (h_r_) or underconsumed (l_r_) dietary risk factors; ^3^ The specific amount of the dietary risk factor; ^4^ The quantity of the product causing external health effects; ^5^ Health Premium/ Discount of the whole product

**Table 9:** Calculation components, health premiums for sodium, and resulting price increase of selected products in Germany in 2022.

	Product			
	Edeka		Penny-Market	
	Organic Salami (100 g)	Conventional Salami (100 g)	Hochland Gouda (150 g)	San Fabio Mozzarella (220 g)
a_p,cal_^1^ [kcal]	373.00	344.00	510.00	547.80
q_cal_^2^	6.70	7.27	4.90	4.56
m_r,p_^3^ [g]	1.80	1.60	0.90	0.53
m_r,day_^4^ [g]	12.06	11.63	4.41	2.41
Hm_r,day_^5^ [g]	9.76	9.33	2.11	0.11
a_pg,cal,day_^6^ [kcal]	268.76	247.87	235.67	172.59
q_p,day_^7^	0.11	0.10	0.09	0.07
n_r,day_^8^ [g]	1.05	0.92	0.20	0.01
n_r,p_^9^ [g]	1.46	1.28	0.43	0.02
HP_p_^10^	0.0814 €	0.0717 €	0.0241 €	0.0013 €
HP_p_ for 1000 g	0.81 €	0.72 €	0.16 €	0.01 €
Current Price	1.69 €	1.24 €	2.29 €	0.59 €
New Price	1.77 €	1.31 €	2.31 €	0.59 €
Price Increase	4.82%	5.79%	1.05%	0.23%

^1^ Number of kcal of the product containing the dietary risk factor; ^2^ Quantity of the product required to cover the daily requirement; ^3^ Amount of the dietary risk factor contained in the product; ^4^ Harmful per day reference amount of the dietary risk factor contained in the product; ^5^ Average unhealthy amount of the dietary risk factor of the product in the daily consumption; ^6^ Standardised kcal of the product group per day; ^7^ Average unhealthy amount of the dietary risk factor per day; ^8^ Quantity-adjusted unhealthy amount of the dietary risk factor per day; ^9^ Amount of the dietary risk factor of the product in the daily consumption; ^10^ Health Premium/Discount of the product/ingredient

## Data Availability

The data presented in this study are available in the attached MS-Excel spreadsheet. It can be found under the link mentioned at the point “Supplementary Materials”.

## Supplementary Materials

The following supporting information can be downloaded at: https://www.mdpi.com/article/10.3390/nu15153386/s1, Spreadsheet S1: Supplementary Material_Seidel et al. The true price of external health effects from food consumption.
